# Inhibition of SARS-CoV-2 Virus Entry by the Crude Polysaccharides of Seaweeds and Abalone Viscera In Vitro

**DOI:** 10.3390/md19040219

**Published:** 2021-04-15

**Authors:** Sung-Kun Yim, Kian Kim, Inhee Kim, SangHo Chun, TaeHwan Oh, Jin-Ung Kim, Jungwon Kim, WooHuk Jung, Hosang Moon, Bosung Ku, Kyoojin Jung

**Affiliations:** 1 Marine Biotechnology Research Center, Jeonnam Bioindustry Foundation, 21-7, Nonggongdanji 4Gil, Wando-eup, Wando-gun, Jeollanam-do 59108, Korea; kimka3152@jbf.kr (K.K.); csh@jbf.kr (S.C.); sosoth@jbf.kr (T.O.); woohyuki@jbf.kr (W.J.); kjjung@jbf.kr (K.J.); 2 Medical & Bio Decision (MBD) Co. Ltd., #B-8F, 145 Gwanggyo-ro, Yeongtong-gu, Suwon-si, Gyeonggi-do 16229, Korea; ihkim@mbdbiotech.com (I.K.); jungwonk@mbdbiotech.com (J.K.); novagene@mbdbiotech.com (H.M.); goos4684@mbdbiotech.com (B.K.); 3 Marine Bio Team, Wando County, 51, Chonghaejinnam-ro, Wando-eup, Wando-gun, Jeollanam-do 59124, Korea; gadget21c@korea.kr

**Keywords:** COVID-19, SARS-CoV-2, seaweed, abalone viscera, polysaccharide, antiviral activity, antioxidant

## Abstract

Much attention is being devoted to the potential of marine sulfated polysaccharides as antiviral agents in preventing COVID-19. In this study, sulfated fucoidan and crude polysaccharides, extracted from six seaweed species (Undaria pinnatifida sporophyll, Laminaria japonica, Hizikia fusiforme, Sargassum horneri, Codium fragile, Porphyra tenera) and Haliotis discus hannai (abalone viscera), were screened for their inhibitory activity against SARS-CoV-2 virus entry. Most of them showed significant antiviral activities at an IC50 of 12~289 μg/mL against SARS-CoV-2 pseudovirus in HEK293/ACE2, except for P. tenera (IC50 > 1000 μg/mL). The crude polysaccharide of S. horneri showed the strongest antiviral activity, with an IC50 of 12 μg/mL, to prevent COVID-19 entry, and abalone viscera and H. fusiforme could also inhibit SARS-CoV-2 infection with an IC50 of 33 μg/mL and 47 μg/mL, respectively. The common properties of these crude polysaccharides, which have strong antiviral activity, are high molecular weight (>800 kDa), high total carbohydrate (62.7~99.1%), high fucose content (37.3~66.2%), and highly branched polysaccharides. These results indicated that the crude polysaccharides from seaweeds and abalone viscera can effectively inhibit SARS-CoV-2 entry.

## 1. Introduction

Since the first identified case in December 2019, COVID-19, caused by the SARS-CoV-2 virus, has spread worldwide, causing tremendous fear and a long recession. Approximately 119 million cases of COVID-19 and more than 2.65 million deaths have been reported [1]. On 11 and 18 December 2020, and 27 February 2021, respectively, the U.S. Food and Drug Administration issued an emergency use authorization (EUA) for the Pfizer–BioNTech (indicated for individuals aged 16 years or older), Moderna, and Janssen (both vaccines are indicated for individuals aged 18 years or older) COVID-19 vaccines for the prevention of coronavirus disease 2019 (COVID-19) caused by severe acute respiratory syndrome coronavirus 2 (SARS-CoV-2) [2]. However, a total of eight (Pfizer–BioNTech, Moderna, Janssen, AstraZeneca–Oxford, Sinovac Biotech, Gamaleya, CanSino Biologics, and Sinopharm) vaccines are now available for public use, in limited quantities, in at least 122 countries [3]. To date, at least 359 million doses of coronavirus vaccines have been administered around the world [3,4]. According to Bloomberg, the latest vaccination rate is an average of 9,245,747 doses per day worldwide, and they estimate that it will take another 3.3 years to cover 75% of the population with a two-dose vaccine at the current rate [3,4]. Although vaccination has begun worldwide, it will take a long time to safely achieve herd immunity against COVID-19 by vaccine, so effective therapies are still needed to prevent or treat COVID-19.

Seaweeds are an excellent source of bioactive compounds such as polysaccharides, dietary fibers, amino acids, essential fatty acids, carotenoids, phlorotannins, vitamins, and minerals [5,6]. These compounds have been reported to have a variety of pharmacological activities such as antitumor, antiviral, antioxidant, antimicrobial, anticoagulant, and immune-inflammatory effects [7]. Kwon et al. [8] reported that sulfated polysaccharides, such as sulfated fucoidans (RPI-27; MW~100 kDa and RPI-28; MW~12 kDa) extracted from the seaweed *Saccharina japonica*, have strong antiviral activity against SARS-CoV-2 in vitro. It has been suggested that the sulfated polysaccharide could bind to the viral spike glycoprotein, inhibiting virus entry into the host cell. Fucoidan has also been shown to have antiviral activity against influenza A virus, hepatitis B virus, canine distemper virus, and human immunodeficiency virus (HIV) in vitro [9,10]. In addition, a recent review by Fitton et al. [10] mentioned that fucoidans have potential as supplementary agents to attenuate damage subsequent to respiratory viral infections by restoring innate immune function and inhibiting inflammation. Phlorotannins also exert antiviral effects against the influenza virus, HIV, and porcine epidemic diarrhea coronavirus [11]. Significantly, with the exception of the monomeric phloroglucinol, phlorotannins inhibit SARS-CoV 3CL^pro^. Among the phlorotannins, dieckol has the most potent inhibitory activity against SARS-CoV 3LC^pro^. SARS-CoV-2 3CL^pro^ is very similar to SARS-CoV 3CL^pro^; thus, phlorotannins are expected to inhibit SARS-CoV-2 3CL^pro^ as well [12,13].

Abalone is one of the most highly valued seafoods in the world and is widely cultivated in South Korea, China, Japan, and Southeast Asian countries. Abalone viscera, which are considered byproducts of abalone processing, account for approximately 15–25% of the total weight of abalone [14]. During the ocean-rearing period, abalones are primarily fed various seaweed, including *Ecklonia maxima*, *Laminaria japonica*, *Ulva rigida*, *Carpoblepharis flaccida*, *Gracilaria gracilis*, and *Ulva lactuca* [15,16,17,18]. In South Korea (Wando), the majority of *Haliotis discus hannai* is co-cultured with kelp (*L. japonica*). *Laminaria japonica* is the most common and often the only food source for abalone. Therefore, it is expected that the visceral portion of abalone may contain concentrated nutritional components such as polysaccharides (approximately 18%), glycoproteins, and other compounds with various biological activities, including free radical scavenging, antioxidant, antibacterial, anti-tumor, and antiviral activities [16,17,18,19].

In this study, we investigated the inhibitory activities of eight crude polysaccharides (CPs), including fucoidan and the crude polysaccharides from *Undaria pinnatifida* sporophyll (CPUP), *Laminaria japonica* (CPLJ), *Hizikia fusiforme* (CPHF), *Sargassum horneri* (CPSH), abalone viscera (CPAV), *Codium fragile* (CPCF), and *Porphyra tenera* (CPPT) against the interaction between SARS-CoV-2 S-glycoprotein (using a SARS-CoV-2 pseudovirus) and ACE2.

## 2. Results

### 2.1. Composition Analysis of Crude Polysaccharides

Analyses of composition were performed by measuring total carbohydrates, protein, sulphate ions, and phenolic content in order to determine the properties of the crude polysaccharides (CPs). These results are presented in Table 1. The total carbohydrate contents in the CPs ranged widely from 29.0 to 99.1%, and among them, the crude polysaccharide from *Sargassum horneri* (CPSH) had the highest content (99.1%), followed by *Hizikia fusiforme* (CPHF, 94.4%), abalone viscera (CPAV, 62.7%), *Undaria pinnatifida* sporophyll (CPUP, 60.3%), fucoidan (58.6%), *Laminaria japonica* (CPLJ, 54.6%), *Porphyra tenera* (CPPT, 46.0%), and *Codium fragile* (CPCF, 29.0%), respectively. Total protein content was analyzed by BCA (bicinchoninic acid) assay [20]. The highest level of total protein content was found in CPAV (22.3%), followed by CPHF (10.9%), CPLJ (8.7%), CPSH (4.0%), CPUP (2.6%), CPCF (1.4%), and fucoidan (0.5%), respectively. Protein was not detected in CPPT. The total sulfate content was determined by the BaCl_2_–gelatin turbidity method [21]. CPUP had the highest sulfate ion content (36.3%), followed by fucoidan (30.7%), CPHF (20.4%), CPCF (16.8%), CPSH (9.8%), and CPAV (0.5%), respectively. CPPT and CPLJ were almost completely sulfate ion-free and were not detected. The total phenolic content of the CPs was measured according to the Folin–Ciocalteu method [22]. The total phenolic content was low in all CPs, and among them, the highest level was in CPHF (1.09%), followed by CPSH (1.03%), CPAV (0.43%), CPUP (0.15%), CPLJ (0.14%), CPPT (0.12%), and fucoidan (0.04%), respectively. In CPCF, phenolic compounds were not detected.

### 2.2. Monosaccharide Composition of Crude Polysaccharides

The monosaccharide compositions of the crude polysaccharides were analyzed by HPLC analysis based on precolumn PMP derivatization as shown in Table 2. The fucose content was much higher than that of any other monosaccharide in all CPs except for CPCF and CPPT. The highest level of total fucose content was found in CPSH (66.2%), followed by CPUP (50.3%), CPHF (41.8%), CPAV (37.3%), CPLJ (22.1%), fucoidan (12.8%), and CPCF (2.6%), respectively. Galactose content was highest in CPPT (32.4%), followed by CPUP (30.7%), CPAV (12.9%), CPLJ (10.2%), CPHF (9.6%), fucoidan (7.9%), CPSH (6.9%), and CPCF (6.6%), respectively. Mannose and Rhamnose contents were the highest in CPHF (16.6 and 11.7%, respectively). Arabinose was not detected in any CP and xylose was not detected in fucoidan.

### 2.3. Trace Elements in the Crude Polysaccharides

Results for the analysis of minerals and trace element concentrations in the CPs are outlined in Table 3. In all samples, Ca, K, Mg, and Na were the most abundant elements and Cd, Ga, Li, Pb, and Sr were not detected. Some mineral and trace elements (K > B > Ti > In > Fe > Al > Cr > Zn > Ba > Ni) were highest in CPPT, whereas, As, Bi, and Cu were not detected. Titanium (Ti, 19,386.2 μg/g of sample) and nickel (Ni, 39.4 μg/g) were found only in CPPT. Calcium (Ca), sodium (Na), and manganese (Mn) were the highest in CPCF (282,637.1 μg/g, 94,248.6 μg/g, 343.2 μg/g, respectively) compared to other CPs, but Ca, Na, and Mn were lowest in CPPT (62,308.7 μg/g), CPSH (424.7 μg/g), and CPUP (1.1 μg/g), respectively. Arsenic (As) was detected in small concentrations (ranging from 1.9 to 33.1 μg/g) in all CPs except for CPCF, fucoidan, and CPPT. Magnesium (Mg), silver (Ag), and bismuth (Bi) were the most abundant in CPLJ (17,807.9 μg/g, 926.3 μg/g, and 740.1 μg/g, respectively) but potassium (K) was the lowest (876.0 μg/g) compared to other CPs.

### 2.4. Molecular Weight Distribution of Crude Polysaccharides

**Figure 1:**
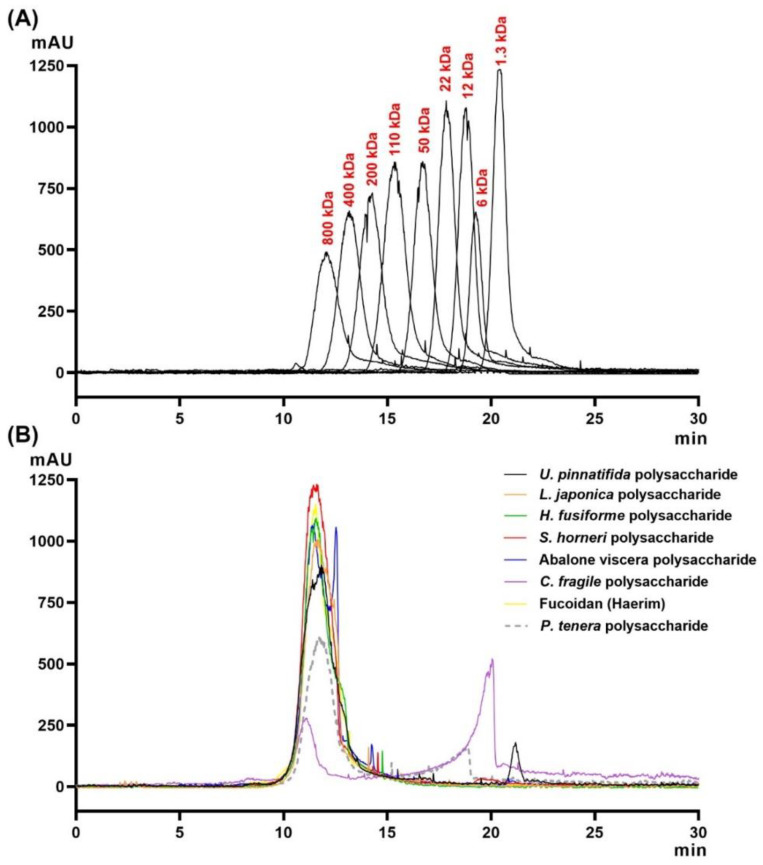
Gel permeation chromatogram for 10 mg/mL of the crude polysaccharides from seaweeds and abalone viscera: (**A**) pullulan samples of molecular weight 1.3, 6, 12, 22, 50, 110, 200, 400, and 800 kDa were used as standards; (**B**) fucoidan and the crude polysaccharides from seaweed and abalone viscera were analyzed by gel permeation chromatography (GPC) using an Ultrahydrogel 500 column. The mobile phase was water. An evaporative light scattering detector (ELSD) was used, the injection volume was 20 μL, and the flow rate was 0.5 mL/min at 45 °C.

Molecular weight (MW) was estimated using gel permeation chromatography (GPC) and the MW distribution is presented in Figure 1. The major and minor peaks of all CPs were detected around 11 and 20 min, respectively. Among the CPs, CPSH displayed the highest peak with a retention time around 11 min, followed by fucoidan, CPHF, CPAV, CPLJ, CPUP, CPPT, and CPCF, respectively. However, CPCF displayed the highest peak with a retention time of around 20 min, and CPPT and CPUP were detected around 19 and 21 min, respectively. Moreover, in CPAV, a different peak that was not present in the seaweed polysaccharide results was detected at around 12 min. The average polysaccharide molecular weight in the CPs was over 800 kDa, and some CPs were distributed in ranges of 1~10 kDa when compared to pullulan standard peaks. The molecular weight of fucoidan was also over 800 kDa.

### 2.5. Cytotoxicity Assay

**Figure 2:**
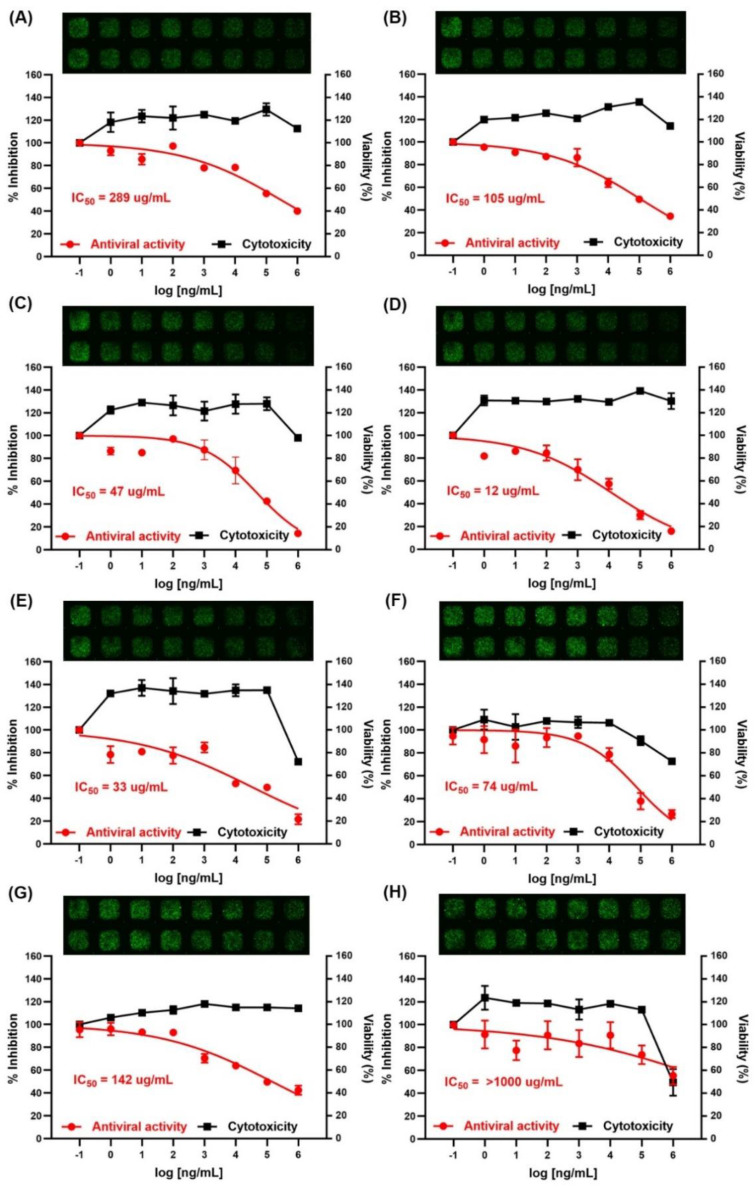
Determination of the cytotoxicity (right y axis, black squares) and antiviral activity (left y axis, red circles) of the crude polysaccharides in HEK293/ACE2 cells. The viability of HEK293/ACE2 cells was assessed using an CellTiter-Glo^®^ Luminescent cell viability assay kit (Promega, Madison, WI, USA) after treatment with the indicated concentrations of the crude polysaccharides from (**A**) *Undaria pinnatifida* sporophyll, (**B**) *Laminaria japonica*, (**C**) *Hizikia fusiforme*, (**D**) *Sargassum horneri*, (**E**) abalone viscera, (**F**) *Codium fragile*, (**G**) fucoidan (Herim), and (**H**) *Porphyra tenera* for 96 h. The inhibition of viral infection by the crude polysaccharides was performed with a SARS-CoV-2 pseudovirus (COV-PS02). Results are expressed as a percent of inhibition in drug-treated cultures versus untreated and were plotted with Graphpad prism software (Graph-Pad, San Diego, CA, USA). Values are the means ±  S.D. (*n* = 3). The viral infection of HEK293/ACE2 cells was detected as a GFP fluorescence by an MBD ASFA scanner (MBD Biotech., Swon, South Korea) and was presented on the top of each graph.

The viability of HEK293/ACE2 cells was assessed using a Cell Titer-Glo Luminescent cell viability assay kit (Promega, Madison, WI, USA). When the HEK293/ACE2 cells were treated with each CP at the final concentration range from 1 ng/mL to 1 mg/mL (serial diluted 1/10) for 96 h, most of the CPs did not show cytotoxicity (Figure 2). However, CPAV, CPCF, and CPPT were slightly cytotoxic at a concentration of 1 mg/mL. CPAV showed notable cytotoxicity when applied at a concentration of 1 mg/mL, following a degree of cytotoxicity by CPPT, CPCF, CPHF, CPLJ, CPUP, CPSH, and fucoidan. Nevertheless, the CC50 of all CPs was over 500 μg/mL. When the cells were treated with CP, the cell growth tended to increase temporarily at low concentrations of all CPs.

### 2.6. Inhibition of Viral Infection

The testing of the inhibition of viral infection by CPs was performed with a SARS-CoV-2 pseudovirus (COV-PS02; Creative Diagnostics, Shirley, NY, USA). As shown in Figure 2, all CPs, except CPPT, inhibited SARS-CoV-2 pseudovirus infection of HEK293/ACE2 cells at various concentrations. After viral exposure, the infection rate was determined by GFP (green fluorescent protein) fluorescence imaging, which is represented at the top of Figure 2. Through a decrease in GFP, it was observed that viral infection typically decreased with the increasing concentration of CP. Among the CPs tested, CPSH showed the strongest antiviral activity with an IC50 of 12 μg/mL, followed by CPAV (33 μg/mL), CPHF (47 μg/mL), CPCF (74 μg/mL), CPLJ (105 μg/mL), fucoidan (142 μg/mL), and CPUP (289 μg/mL), respectively.

## 3. Discussion

The SARS-CoV-2 virus can enter a host cell by the interaction of the COVID-19 spike glycoprotein and the ACE2 receptor or another receptor, neuropilin-1 (NRP1), of the host cell [23,24]. In previous studies, sulfated polysaccharides were reported to effectively inhibit SARS-CoV-2 entry by interfering with the interaction of the spike protein with the ACE2 receptor of the host cell in vitro [8,25,26]. Based on these studies, we chose various edible seaweeds (*U. pinnatifida*, *L. japonica*, *H. fusiforme*, *S. horneri*, *C. fragile*, and *P. tenera*) known to be high in sulfated polysaccharides and the viscera of the abalone, which eats such seaweeds, and extracted the crude polysaccharides after removing alginic acid with CaCl_2_. Seaweeds are known to produce different types of polysaccharides (laminaran, alginate, carrageenan, agar, porphyran, xylan, mannitol, fucoidan, etc.) and contain numerous components that can exert antioxidant, anti-inflammatory, and antiviral effects [7,10].

As SARS-CoV-2 is highly infectious and pathogenic, we used a SARS-CoV-2 pseudovirus (COV-PS02, Creative Diagnostics) to investigate whether fucoidan or the CPs of seaweeds and abalone viscera could inhibit host cell infiltration, through interaction with the COVID-19 spike glycoprotein and the ACE2 receptor of the host cell in vitro. Most of the CPs of seaweeds and CPAV effectively inhibited SARS-CoV-2 entry, like the previously reported sulfated polysaccharides [8,25,26]. None of the CPs showed toxicity below 1 mg/mL of CPs. Among the tested polysaccharides, CPSH showed the strongest antiviral activity with an IC50 of 12 μg/mL to prevent COVID-19 entry. CPAV and CPHF could also inhibit SARS-CoV-2 infection with an IC50 of 33 μg/mL and 47 μg/mL, respectively. These CPs (from *S. horneri*, abalone viscera, and *H. fusiforme*) have common properties such as high molecular weight (>800 kDa), high total carbohydrate (99, 63, and 94%, respectively), and higher fucose content (66, 37, and 42%, respectively) than other CPs. However, the sulfate ion content (9.8, 0.5, and 20.4%, respectively) of these CPs was somewhat lower than that of others (fucoidan and CPUP had over 30% sulfate ion content). These results indicate that the molecular weight, total polysaccharide content, and fucose content were important factors in the inhibitory effect of the CPs against SARS-CoV-2 host cell entry. According to previous studies [8,25], RPI-27, a high molecular weight (~100 kDa), branched polysaccharide from *Saccharina japonica,* was shown to have higher antiviral activity (EC50 = 8.3 μg/mL) than RPI-28 (EC50 = 16 μg/mL), which is a lower molecular weight (~12 kDa). Sulfated galactofucan (195.0 kDa) and glucuronomannan (7.0 kDa) strongly inhibited interaction between the SARS-CoV-2 S-glycoprotein and heparin (IC50 values are 27 and 231 nM, respectively).

Interestingly, according to our present results, CPAV also displayed high antiviral activity compared to the CPs from seaweeds. The CPAV was lower in total carbohydrate, sulfate ion, and fucose content than CPSH and CPHF, while the galactose and rhamnose contents were higher than the same two CPs. In gel permeation chromatography, a different peak in CPAV was detected at around 12 min compared to other CPs. Abalones are primarily fed various seaweeds including *E. maxima*, *L. japonica*, *U. rigida*, *C. flaccida*, *G. gracilis*, and *U. lactuca* for the ocean-rearing period [15,16,17,18] and these seaweeds are broken down by commensal marine microorganisms and digestive enzymes in viscera. In this present study, the abalone viscera came from 5-year-old abalone raised with L. japonica produced by Dashimachonbok Fisheries (Wando, Jeollanam-do, South Korea). The monosaccharide composition of CPAV was similar to CPLJ, but the CPAV displayed higher antiviral activity (IC50 = 33 μg/mL) than CPLJ (IC50 = 105 μg/mL). The reason is thought to be that the CPAV had higher total carbohydrate, fucose, and galactose content, and the amount of high molecular weight polysaccharides was higher than the CPLJ. Abalone viscera is composed of polysaccharides, peptides, and other bioactive molecules with various biological activities, including free radical scavenging, antioxidant, antibacterial, anti-tumor, and immunomodulatory activities [19,27,28,29]. It is presumed that the synergistic effect of these bioactive compounds and high molecular weight polysaccharides resulted in the strong antiviral activity.

Unlike previous studies, fucoidan (Haerim) showed relatively low antiviral activity (IC50 = 142 μg/mL) compared to other CPs except for CPUP (IC50 = 289 μg/mL) and CPPT (IC50 > 1000 μg/mL). It has a high (30.7%) sulfate ion content, but the antiviral activity is lower than that of CPLJ (IC50 = 105 μg/mL) which contains a similar total carbohydrate content but a lower sulfate ion content. Even CPCF, with low molecular weight polysaccharides, displays higher antiviral activity (IC50 = 74 μg/mL) than fucoidan. The reason is that the fucoidan used in this study was extracted and purified from *U. pinnatifida* sporophyll, while the sulfated fucoidan used in previous studies was extracted from *S. japonica*. A key difference between the fucoidan from *U. pinnatifida* and that from other brown seaweed species such as *S. japonica* or *S. horneri* is the backbone of the sulfated polysaccharide molecule. SPSJ is a highly branched, partially acetylated, sulfated galactofucan, built up of (1→3)-α-L-fucose residues, while SPUP is a partially acetylated, highly sulfated galactofucan consisting of (1→3)- or (1→3); (1→4)-α-l-fucose residues [30]. Meanwhile, SPSH is established to consist mostly of the repeating → 3-α-l-Fuc*p*(2SO_3_^−^)-1 → 4-α-l-Fuc*p*(2,3SO_3_^−^)-1 → fragment with insertions of the →3-α-l-Fuc*p*(2,4SO_3_^−^)-1 → fragment and unsulfated side chains with the α-L-Fuc*p*-1 → 2-α-l-Fuc*p*-1 → structure connected to the main one at C4 of the monosaccharide residue [31]. SPCF is composed of highly sulfated 3-linked β-d-galactopyranose and β-l-arabinopyranose residues [32]. The backbone of the SP molecule and its branches appear to play an important role in inhibiting SARS-CoV-2 virus entry.

## 4. Materials and Methods

### 4.1. Chemicals and Reagents

d-mannose, d-galactose, d-xylose, l-rhamnose, d-glucose, d-arabinose, d-fucose, d-fructose, calcium chloride anhydrous, sodium phosphate dibasic, sodium hydroxide, barium chloride anhydrous, 1-Phenyl-3-methyl-5-pyrazolone (PMP), Folin–Denis’ reagent, gallic acid, trifluoroacetic acid (TFA), trichloroacetic acid (TCA), phenol, chloroform, sulfuric acid, nitric acid, hydrogen chloride (1N), hydrogen peroxide (30%), gelatin, copper(II) sulfate solution (4%, *w*/*v*), and bicinchoninic acid were purchased from Sigma-Aldrich (St. Louis, MO, USA). Bovine serum albumin standard was purchased from Thermo Fisher Scientific (Waltham, MA, USA). All analytical grade organic solvents (acetonitrile, ethanol, methanol, deionized water) were purchased from Burdick & Jackson chemicals (Muskegon, MI, USA). Ultra-pure argon (99.99%), nitrogen (99.99%), and carbon dioxide were supplied from Daechang Gas (Songha-dong, Gwangju, South Korea).

### 4.2. Seaweed Collection

The algae Laminaria japonica, Hizikia fusiforme, sporophyll of Undaria pinnatifida, Sargassum horneri, Porphyra tenera, and Codium fragile were cultured in Wando, Jeollanam-do, South Korea. Laminaria japonica, Hizikia fusiforme, and sporophyll of Undaria pinnatifida were collected in May, 2020, and Porphyra tenera was collected in January, 2020, and Sargassum horneri and Codium fragile were collected in August, 2020. The fresh seaweed (20 kg) was immediately washed with tap water in order to remove salt, epiphytes, and sand attached to the surface of the samples and then dried. The fresh abalone (Haliotis discus hannai) viscera were purchased from Dashimachonbok (Wando, jeollanam-do, South Korea), washed, and dried by lyophilization. The dried seaweed and abalone viscera were crushed, ground into a powdered form, passed through a 100-mesh sieve, and then stored at −20 °C. Fucoidan was obtained from Haerim Fucoidan (Wando, Jeollanam-do, South Korea).

### 4.3. Preparation of the Crude Polysaccharides

Each dried powder (2 kg) of seaweed and abalone viscera was treated twice at 100 °C with 40 L of water for 6 h, and the hot solution (80 L) was separated from the algae residues by successive filtration through cotton wool. The extracts were concentrated to about 4L under reduced pressure and then 2% CaCl_2_ and 1.2 L of 85% (*v*/*v*) ethanol were added to eliminate the alginate. The extracts were centrifuged at 10,000 g and the supernatants were filtered through Whatman’s filter papers (150 mm, 541). The filtered solution was dialyzed against distilled water and concentrated to about 1L using an ultrafiltration hollow fiber cartridge (10,000 NMWC, 31.8 L × 3.2 cm O.D., Cytiva, Marlborough, MA, USA). Finally, the CP was lyophilized and stored at −20 °C.

### 4.4. Total Carbohydrate Content

The total sugar content was analyzed using the phenol−sulfuric acid method [33]. D-glucose was used as the standard. Serially diluted standards were prepared at 20, 40, 80, 160, 320, and 640 μg/mL. Two hundred microliters of eluted CPs or eluted standards were transferred into 10 mL test tubes containing 0.5 mL of 5% phenol, followed by the addition of 2.5 mL of 18 M H_2_SO_4_. Each tube was mixed thoroughly and incubated for 30 min. The absorbance was measured at 480 nm with a Varioskan LUX (Thermo Fisher Scientific, Waltham, MA, USA). Serially diluted standards were calculated to obtain the standard curve.

### 4.5. Determination of Sulfate Contents

The sulfate content of CPs was determined by the BaCl_2_–gelatin turbidity method [21]. Gelatin solution (0.5%) was prepared in hot water (60~70 °C) and stored at 4 °C overnight. Barium chloride (2 g) was dissolved in the gelatin solution and allowed to stand for 2~3 h at 25 °C. To the CP solution (0.2 mL), 3.8 mL of 4% trichloroacetic acid was added, followed by 1 mL of the BaCl_2_–gelatin solution, and the absorbance was measured at 360 nm by UV spectrophotometer (Thermo Fisher Scientific, Waltham, MA, USA) after incubation for 20 min at room temperature. A standard curve was prepared with a potassium sulfate solution between approximately 20 and 200 μg/mL.

### 4.6. Total Phenolic Content Analysis

The determination of total phenolics was carried out spectrophotometrically using Folin–Denis’ reagent [22]. Briefly, 0.5 mL of CP or standard (gallic acid) were diluted in 0.5 mL of water. Thereafter, 0.5 mL of Folin–Denis’ reagent was added and let stand for 5 min. Finally, 0.5 mL of saturated Na_2_CO_3_ solution was added, and after 30 min the absorbance was measured at 700 nm with a Varioskan LUX (Thermo Fisher Scientific, Waltham, MA, USA). Gallic acid was used as the standard prepared in different concentrations ranging from 0 to 100 μg/mL.

### 4.7. BCA Assay

The CPs were prepared at 2 mg/mL Each sample (10 µL) was added to a 96-well plate in triplicate, and deionized water was added to bring the total volume to 20 µL. Blanks were prepared with 20 µL of deionized water in triplicate. Fresh BCA solution and 4% copper solution were prepared according to the manufacturer’s instructions, and 180 μL was added to each well, which were mixed by shaking and then incubated at 37 °C for 30 min. A standard curve was made with bovine serum albumin (BSA; 0, 0.02, 0.04, 0.06, 0.08, and 0.1 mg/mL), and the absorbance was read at 562 nm (A_562nm_) with a Varioskan LUX (Thermo Fisher Scientific, Waltham, MA, USA).

### 4.8. Monosaccharide Analysis of Crude Polysaccharides

Each CP (0.1 g) was treated with 50 mL of 2 M trifluoroacetic acid at 110 °C for 8 h. After the hydrolysis of the polysaccharide, 1 mL of reaction medium was dried with a vacuum concentrator, and 450 µL of 0.3 M NaOH was added to resuspend the sample. The PMP derivatization of monosaccharides was carried out according to a previous method [34] with minor modifications. The hydrolyzed samples were mixed with 450 µL of 0.5 M PMP solution (in methanol). The mixtures were incubated at 70 °C for 30 min in a water bath, then cooled to room temperature and neutralized with 450 µL of 0.3 M HCl. Chloroform (1.0 mL each) was added and mixed, then the organic phase was carefully removed. The extraction process was repeated three times and the aqueous layer was passed through a 0.45 µm syringe filter before HPLC analysis. Standard solutions of the eight monosaccharides (arabinose, fucose, galactose, glucose, mannose, rhamnose, and xylose; 20 mM) were also treated as described above. The chromatographic analysis was performed on an LC-20AD HPLC system (Shimadzu, Kyoto, Japan) consisting of a binary pump (LC20AD XR; Shimadzu, Japan), an automatic injection pump (SIL-20AC XR Prominence Autosampler; Shimadzu, Japan), a degasser, a column oven controller, and a photodiode array detector (PDA; Shimadzu, Japan). The monosaccharides were separated on a reverse-phase Sunfire C_18_ column (5 μm particle size, 250 × 4.6 mm I.D., Waters, Milford, MA, USA) coupled to a C_18_ guard column (5 μm particle size, 15 × 4.6 mm I.D.). The injection volume was 20 µL with a flow rate of 1.0 mL/min at 25 °C. Mobile phase A was 40% acetonitrile with 0.05 M KH_2_PO_4_ (pH 6.9) and mobile phase B was 15% acetonitrile with 0.05 M KH_2_PO_4_ (pH 6.9); gradient elution was performed at 92–83–92% B at 0–10–40 min, respectively. Each sample was analyzed in duplicate.

### 4.9. Analysis of Minerals and Trace Elements

The crude polysaccharides were acid-digested using Sineo Microwave Digestion (MDS-8, Shanghai Sineo Microwave Chemistry Technology, Shanghai, China). Briefly, 0.500 g of each crude polysaccharide was digested with nitric acid (Supra Pure Metal 65%). Approximately 10 mL of nitric acid was added to a Teflon vessel and 0.500 g of each crude polysaccharide was allowed to predigest for 20 min before 4 mL of nitric acid and 1 mL of hydrogen peroxide (30%) were added. The pre-digested seaweed samples were capped. The Teflon vessels and their contents were subjected to microwave digestion, which was operated at four steps of temperature: 130 °C for 10 min, 150 °C for 5 min, 180 °C for 5 min, and a holding temperature of 200 °C for 10 min. At the end of the digestion, all seaweed samples were allowed to cool at room temperature. Then, the digests inside the vessels were quantitatively transferred into 50 mL polyethylene centrifuge vials. Digested samples were analyzed by Agilent 700 Series ICP-OES (Agilent 710ES, Agilent technologies Inc., Santa Clara, CA, USA) equipped with ICP Expert II software (Agilent 710-ES instrument Software, version 2.0). The instrument parameters were set as follows: 1.35 kW of radio frequency power, 15 L/min of plasma gas flow rate, 1.5 L/min of auxiliary argon flow rate, 160 kPa of nebulizer pressure, 5 s of replicate read time, 10 rpm peristaltic pump rate, 40 s of sample uptake delay, 10 s of rinse time, SPS3 sampler, cyclonic chamber, and seaspray nebulizer. The certified reference material, ICP multi-element standard solution IV (MES-04-1, AccuStandard, New Haven, CT, USA) was used as a standard reference material. Calibration standards were prepared from a multi-element standard solution of 10 mg/L. According to the manufacturer, the limit of detection was as follows: Ag = 0.44 μg/L; Al = 4.62 μg/L; As = 4.13 μg/L; B = 1.65 μg/L; Ba = 0.06 μg/L; Bi = 18.8 μg/L; Ca = 1.0 μg/L; Cd = 0.49 μg/L; Co = 3.0 μg/L; Cr = 1.78 μg/L; Cu = 2.02 μg/L; Fe = 0.64 μg/L; Ga = 2.85 μg/L; In = 4.95 μg/L; K = 17.7 μg/L; Li = 0.02 μg/L; Mg = 0.01 μg/L; Mn = 0.03 μg/L; Na = 2.23 μg/L; Ni = 4.21 μg/L; Pb = 8.52 μg/L; Sr = 0.04 μg/L; Ti = 0.02 μg/L; and Zn = 0.64 μg/L. All analyses were repeated at least three times.

### 4.10. Estimation of Average Molecular Weights

The molecular size distribution of the various CPs from seaweeds and abalone viscera was determined by gel permeation chromatography (GPC) using a LC-20AD HPLC system (Shimadzu, Kyoto, Japan) consisting of a binary pump (LC20AD XR; Shimadzu, Japan), an automatic injection pump (SIL-20AC XR Prominence Autosampler; Shimadzu, Japan), a degasser, a column oven controller, and an evaporative light scattering detector (ELSD; Shimadzu, Japan). An Ultrahydrogel 500 (300 × 7.8 mm) column (Waters Co., Miliford, MA, USA) in combination with a guard column (Ultrahydrogel, 6 × 40 mm, Waters, Milford, MA, USA) was used to maximize the resolution. Standard pullulans including 1300, 6000, 12,000, 22,000, 50,000, 110,000, 200,000, 400,000, and 800,000 Da were used as molecular mass markers. The injection volume was 20 µL and eluted with distilled water at 45 °C with a flow rate of 0.5 mL/min. Each sample was analyzed in duplicate.

### 4.11. SARS-CoV-2 Pseudovirus and Cell

SARS-CoV-2 Pseudovirus (COV-PS02), expressing S-glycoprotein on the surface of the Lentivirus, and HEK293/ACE2 cells, genetically engineered to overexpress angiotensin-converting enzyme 2, were purchased from Creative Diagnostics (Shirley, NY, USA). Dulbecco’s modified Eagle’s medium (DMEM), fetal bovine serum (FBS), and geneticin (G-418 sulfate) were purchased from Thermo Fisher Scientific (Waltham, MA, USA). The Cell Titer-Glo Luminescent cell viability assay kit was purchased from Promega (Madison, WI, USA). HEK293/ACE2 was maintained at 37 °C, 5% CO_2_ in Dulbecco’s modified Eagle medium (DMEM) supplemented with 10% (*v*/*v*) heat-inactivated fetal bovine serum and 0.5 mg/mL of G418. The cells were sub-cultured within 48 h intervals.

### 4.12. Cytotoxicity Assay

Assays of the cytotoxicity of the crude polysaccharides were performed using a Cell Titer-Glo Luminescent cell viability assay kit. After the HEK293/ACE2 cells were treated with each 20 µL sample of crude polysaccharides at various concentrations (10-fold serial dilution in range of 1 ng/mL to 1 mg/mL, final D.W 5%) for 96 h, 4 µL/well of Cell titer Glo reagent was added and the vessel was shaken for 2 min at 700 rpm. After 10 min, the mixture was read with a luminometer and cell viability was calculated as follows:

(1) $$Cell\;Viability\;\left(\%\right)=\frac{RLU\;sample}{RLU\;conc.}\times 100$$

where the RLU sample is the luminescence of the experimental sample and RLU conc. is the luminescence of the control. Cytotoxicity was calculated as follows:

(2) Cytotoxicity (%)=100−% Cell Viability

The 50% cytotoxic concentration for crude polysaccharides was determined using nonlinear regression analysis with Graph-Pad Prism software (Graph-Pad, San Diego, CA, USA). Each sample was analyzed in duplicate wells and repeated three times.

### 4.13. Inhibition of Viral Infection

To investigate the inhibitory effect of the crude polysaccharides on viral infection, HEK293/ACE2 was seeded in 384 well plates at a concentration of 2 × 10^3^ cells/well using DMEM and incubated at 37 °C, 5% CO_2_ for 24 h. When HEK293/ACE2 cells had grown to a density of 30~40% in the 384-well plate, the cells were treated with a mixture of 20 μL of the particular crude polysaccharides (serial diluted 1/10 in a concentration range of 1 ng/mL to 1 mg/mL, final D.W 5%) and 1 μL of SARS-CoV-2 pseudovirus (titer: 1.0E + 07 TU/mL) to induce viral infection. After 96 h incubation, the viral infection rate was analyzed by scanning GFP fluorescence with an MBD ASFA scanner (MBD Biotech., Suwon, South Korea). To calculate the cell penetration inhibition of the SARS-CoV-2 pseudovirus, the GFP fluorescence area of infected cells was analyzed with an MBD cell analyzer and was calculated as follows:

(3) $$\%\;inhibition=\frac{\left(GFP\;AREA\;sample\right)}{\left(GFP\;AREA\;conc.\right)}\times 100$$

where the GFP AREA sample is the GFP area (μm^2^) of the experimental sample and GFP AREA conc. is the GFP area (μm^2^) of the control. Antiviral activity was calculated as follows:

(4) Antiviral activity (%)=100−% inhibition

Each sample was analyzed in triplicate and the plots were made with Graph-Pad Prism software (Graph-Pad, San Diego, CA, USA).

### 4.14. Statistical Analysis

All experiments were performed in duplicate wells and repeated three times. The area of fluorescent cells infected with the virus was calculated using the MBD Cell Analyzer and the half maximal inhibitory concentrations (IC_50_) were calculated using nonlinear regression analysis of GraphPad Prism 9 software by plotting log (inhibitor) versus normalized response (variable slope). The equation corresponds to:

Y=100/(1+10^((logIC50−X)×HillSlope))

where *Y* is the response, *X* is the logarithm of doses or concentrations, and HillSlope describes the steepness of the family of curves.

## 5. Conclusions

Crude polysaccharides from seaweeds and abalone viscera were screened for antiviral activity against SARS-CoV-2, and most of them showed significant antiviral activity except for CPPT. They can be divided into three groups (strong, medium, and weak inhibition) according to their inhibitory activity. CPSH is alone in the strong inhibition group, CPAV, CPHF, and CPCF are in the medium inhibition group, and CPLJ, CPUP, and fucoidan (Haerim) are in the weak inhibition group, respectively. These bioactive polysaccharides from edible seaweeds and abalone viscera can be examined as therapeutic agents for inhibiting COVID-19 entry through further studies such as the purification and identification of bioactive compounds.

## Figures and Tables

**Table 1:** Total carbohydrate, protein, sulfate ion, and phenolic contents of the crude polysaccharides.

Samples	Total (% of Seaweed Water Extract)			
	Carbohydrate	Protein	Sulfate Ion	Phenolic Content
*Undaria pinnatifida* (sporophyll)	60.3 ± 1.97	2.6 ± 0.50	36.3 ± 1.4	0.15 ± 0.021
*Laminaria japonica*	54.6 ± 1.46	8.7 ± 1.12	* n.d.	0.14 ± 0.070
*Hizikia fusiforme*	94.4 ± 1.82	10.9 ± 0.43	20.4 ± 0.4	1.09 ± 0.030
*Sargassum horneri*	99.1 ± 3.35	4.0 ± 1.14	9.8 ± 0.5	1.03 ± 0.012
Abalone viscera	62.7 ± 4.58	22.3 ± 1.17	0.5 ± 0.1	0.43 ± 0.002
*Codium fragile*	29.0 ± 1.80	1.4 ± 0.44	16.8 ± 1.8	n.d.
Fucoidan	58.6 ± 2.78	0.5 ± 0.31	30.7 ± 0.8	0.04 ± 0.007
*Porphyra tenera*	46.0 ± 1.90	n.d.	n.d.	0.12 ± 0.026

* n.d.: not detected.

**Table 2:** Monosaccharide profiles of the crude polysaccharides.

Monosaccharides (%)	*Undaria pinnatifida* (Sporophyll)	*Laminaria japonica*	*Hizikia fusiforme*	*Sargassum horneri*	Abalone viscera	*Codium fragile*	Fucoidan	*Porphyra tenera*
Mannose	5.7	15.3	16.6	7.6	8.7	4.5	4.3	4.6
Rhamnose	5.9	8.5	* n.d.	n.d.	8.2	5.9	6.5	6.0
Glucose	2.9	3.4	3.1	7.3	3.8	3.6	n.d.	2.8
Galactose	30.7	10.2	9.6	6.9	12.9	6.6	7.9	32.4
Xylose	3.5	4.1	3.7	4.5	3.7	n.d.	* n.d.	3.6
Arabinose	n.d.	n.d.	n.d.	n.d.	n.d.	5.9	6.5	n.d.
Fucose	50.3	22.1	41.8	66.2	37.3	2.6	12.8	4.5

* n.d.: not detected.

**Table 3:** Mineral and trace element contents of the crude polysaccharides.

Mineral (μg g^−1^ of Sample)	*Undaria pinnatifida* (Sporophyll)	*Laminaria japonica*	*Hizikia fusiforme*	*Sargassum horneri*	Abalone viscera	*Codium fragile*	Fucoidan	*Porphyra tenera*
Ag	282.1	926.3	137.2	17.3	15.2	4.8	* n.d.	n.d.
Al	3.5	35.1	28.7	22.6	6.2	74.9	362.5	500.5
As	33.1	18.7	15.4	1.9	29.3	n.d.	n.d.	n.d.
B	1647.1	1098.6	215.9	122.9	65.8	59.8	89.9	23,945.8
Ba	1.8	2.1	1.6	1.8	0.8	4.1	97.4	115.1
Bi	405.7	740.1	577.9	353.2	289.0	470.9	n.d.	n.d.
Ca	95,735.5	136,540.2	80,633.2	107,498.5	68,895.3	282,637.1	82,928.7	62,308.7
Cd	n.d.	n.d.	n.d.	n.d.	n.d.	n.d.	n.d.	n.d.
Co	n.d.	n.d.	n.d.	n.d.	n.d.	n.d.	274.3	118.7
Cr	n.d.	n.d.	1.0	n.d.	0.1	1.4	23.1	273.4
Cu	15.6	44.0	25.5	12.8	14.4	13.9	244.1	n.d.
Fe	25.1	69.8	59.6	103.5	583.1	132.2	216.2	1362.2
Ga	n.d.	n.d.	n.d.	n.d.	n.d.	n.d.	n.d.	n.d.
In	46.0	n.d.	n.d.	n.d.	n.d.	n.d.	1044.3	2183.4
K	45,896.1	876.0	17,531.7	9907.2	6368.1	5456.3	9694.1	91,790.1
Li	n.d.	n.d.	n.d.	n.d.	n.d.	n.d.	n.d.	n.d.
Mg	9143.9	17,807.9	14,562.4	15,518.6	5590.2	6123.8	4221.2	15,042.7
Mn	1.1	2.2	15.0	9.7	1.8	343.2	57.0	157.4
Na	8134.2	3888.1	1068.2	424.7	3597.4	94,248.6	7232.6	27,630.7
Ni	n.d.	n.d.	n.d.	n.d.	n.d.	n.d.	n.d.	39.4
Pb	n.d.	n.d.	n.d.	n.d.	n.d.	n.d.	n.d.	n.d.
Sr	n.d.	n.d.	n.d.	n.d.	n.d.	n.d.	n.d.	n.d.
Ti	n.d.	n.d.	n.d.	n.d.	n.d.	n.d.	n.d.	19386.2
Zn	6.1	59.3	51.0	23.7	26.9	16.3	149.6	258.3

* n.d.: not detected.

## Data Availability

The data presented in this study are available on request from the corresponding author.

## Abbreviations

CP: crude polysaccharide; CPUP: crude polysaccharide from *Undaria pinnatifida* sporophyll; CPLJ: crude polysaccharide from *Laminaria japonica*; CPHF: crude polysaccharide from *Hizikia fusiforme*; CPSH: crude polysaccharide from *Sargassum horneri*; CPAV: crude polysaccharide from abalone viscera; CPCF: crude polysaccharide from *Codium fragile*; CPPT: crude polysaccharide from *Porphyra tenera*; SP: sulfated polysaccharide; SPSJ: sulfated polysaccharide from *Saccharina japonica*; SPUP: sulfated polysaccharide from *Undaria pinnatifida* sporophyll; SPSH: sulfated polysaccharide from *Sargassum horneri*; SPCF: sulfated polysaccharide from *Codium fragile.*
